# Vitamin D receptor gene polymorphisms in atopy

**DOI:** 10.1002/iid3.487

**Published:** 2021-08-03

**Authors:** Laura Tamasauskiene, Ieva Golubickaite, Rasa Ugenskiene, Nikolajs Sjakste, Natalia Paramonova, Lawrence Shih‐Hsin Wu, Lawrence Shih‐Jiu‐Yao Wang, Brigita Sitkauskiene

**Affiliations:** ^1^ Department of Immunology and Allergology Lithuanian University of Health Sciences Kaunas Lithuania; ^2^ Department of Genetics and Molecular Medicine Lithuanian University of Health Sciences Kaunas Lithuania; ^3^ Laboratory of Genomics and Bioinformatics, Faculty of Medicine University of Latvia Riga Latvia; ^4^ Graduate Institute of Biomedical Sciences China Medical University Taichung Taiwan; ^5^ Allergy and Clinical Immunology Research (ACIR) Center, College of Medicine National Cheng Kung University Tainan Taiwan; ^6^ Children's Hospital China Medical University Taichung Taiwan

**Keywords:** atopy, VDR, vitamin D

## Abstract

**Background:**

The occurrence of allergic conditions, for example allergic asthma, rhinitis, and atopic dermatitis, is rising worldwide. These allergic conditions are associated with poor life quality. Vitamin D is proposed to be linked with increased risk and severe forms of allergic diseases.

**Aims:**

This review article aimed to evaluate the vitamin D level role and polymorphisms of vitamin D receptor gene (VDR) in atopy.

**Methods & Materials:**

We analyzed publications that were focusing on levels of vitamin D and/or polymorphism analysis of vitamin D receptor gene in allergic asthma, rhinitis, and atopic dermatitis patients.

**Results:**

We noticed that levels of vitamin D are extensively studied in atopy by many research groups, however, polymorphisms of vitamin D receptor gene and their link with levels of vitamin D lack comprehensive data. There is evidence that vitamin D may be associated with anti‐inflammatory effects in allergic diseases. Some of VDR polymorphisms also may play a role in pathogenesis of these diseases. However, the data from different studies are controversial.

**Discussion:**

The results of different studies are usually inconsistent, most probably due to populational bias or differences in methodology. Even though, more evidence shows a positive impact of vitamin D on the risk and outcomes of allergic diseases, especially atopic dermatitis, and asthma.

**Conclusions:**

There is controversial data about the level of vitamin D and its role in atopy; however, more evidence shows a positive impact on the risk and outcomes of allergic diseases.

## Graphical abstract

Some evidence suggests that vitamin D is linked with increased risk and more severe forms of allergic diseases. Therefore, this review article aimed to evaluate the role of vitamin D level and vitamin D receptor gene (*VDR*) polymorphisms in atopy. We found that vitamin D levels in atopy are extensively studied by many research groups, however, vitamin D receptor gene polymorphisms and their link with vitamin D levels lack comprehensive data. Even though, more evidence shows a positive impact on the risk and outcomes of allergic diseases, especially atopic dermatitis and asthma.

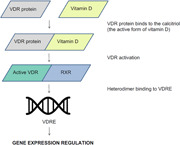

## 1 INTRODUCTION

Traditionally vitamin D is known as a hormone precursor involved in phosphate and calcium metabolism as well as bone growth, but scientists constantly provide new evidence about the benefits of this vitamin for other metabolic pathways of the human body.^1^ New studies focus on the vitamin D impact for the immune system.^1^, ^2^, ^3^

The immune response is a key element in allergic diseases development.^4^, ^5^ The prevalence of such diseases increases worldwide.^5^ The most common atopic conditions are allergic asthma, rhinitis, and atopic dermatitis which are related to poor life quality, social disturbance, and economic burden.^5^, ^6^, ^7^, ^8^ There are some data that low vitamin D level is linked with increased risk and more severe forms of allergic diseases.^9^, ^10^ This vitamin has an impact on the innate and adaptive immune response. Vitamin D3 is transformed to 25(OH)D in the liver and later 25(OH)D is transformed into the active 1,25(OH)2D form in kidneys.^11^ Therefore, it stimulates the vitamin D receptor (VDR), which regulates the expression of genes related with metabolism of calcium, cell proliferation, differentiation, apoptosis, and immunity. The vitamin D receptor is a member of the nucleic receptor superfamily located in dendritic cells (DCs), macrophages, active T cells, and other cells in more than 30 tissues.^12^ Genetic variants of VDR have been studied as a probable factor for autoimmune and allergic diseases as they might affect VDR activity.^13^, ^14^ The aim of this article is to review the literature of vitamin D role and *VDR* gene polymorphisms in atopy.

## 2 THE *VDR* GENE

**Figure 1:**
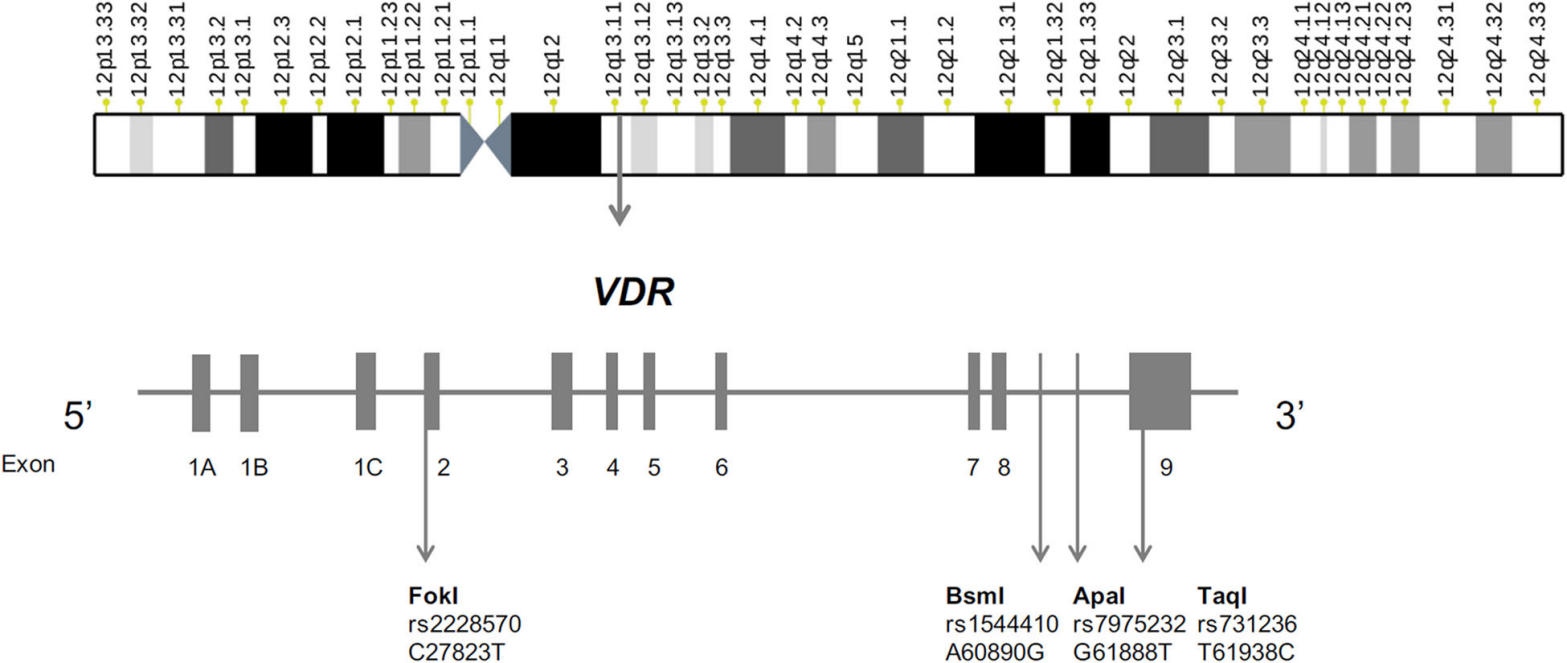
The most common SNPs of the *VDR* gene. SNPs, single‐nucleotide polymorphisms; VDR, vitamin D receptor

**Figure 2:**
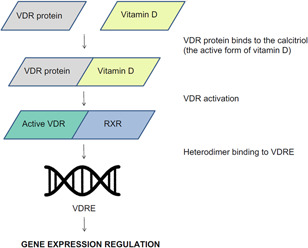
The role of the vitamin D receptor (VDR) protein in gene expression regulation. The VDR protein binds to the calcitriol (the active form of vitamin D) and VDR becomes active. This activation enables VDR to form a complex with retinoid X receptor (RXR). The formed heterodimer then binds to vitamin D response elements (specific regions of DNA) and regulates gene expression of vitamin D responsive genes

The gene of human VDR is located in the 12q13.11 chromosomal area and encodes the vitamin D3 receptor (Figure 1). The biological vitamin D3 activities are regulated by this receptor. Vitamin D3 binds to the calcitriol and allows VDR to partner with the retinoid X receptor (RXR) by forming an RXR/VDR nuclear receptor complex.^15^ This complex is able to bind to certain regions of DNA, called vitamin D response elements, that are located in the promoter region of the gene and are involved in transcription. The complex helps to regulate the activity of vitamin D responsive genes (Figure 2).^16^, ^17^

Single‐nucleotide polymorphisms (SNPs) in the vitamin D3 receptor gene are thought to interfere with vitamin D function and have been studied in various diseases or conditions. The most known and analyzed *VDR* gene SNPs are ApaI, BsmI, FokI, and TaqI. They are also related to clinical outcome. The polymorphisms in ApaI (rs7975232) and BsmI (rs1544410) are in the eighth intron of the vitamin D3 receptor gene. Whereas, SNPs in the FokI (rs2228570) and TaqI (rs731236) are found in the second and nineth exons of the *VDR* gene, respectively. The polymorphisms rs7975232, rs1544410 are associated with decreased stability of messenger RNA and lower levels of expression, while rs2228570 and rs731236 might affect splicing or even structure of the protein.^18^, ^19^

The *VDR* polymorphisms and their associations with allergic diseases were intensively studied by various research groups, however, the results were inconsistent possibly due to differences in methodology or the populational bias.^18^, ^20^

### 2.1 Polymorphisms of *VDR* and levels of vitamin D in asthmatic patients

It is already established that vitamin D plays an important role in asthmatic patients, although, the mechanisms are yet unclear. Experimental research shows that vitamin D suppresses the production of immunoglobulin (Ig) E by and increases the production of interleukin (IL)‐10, suppresses mast cell activation, and reduces pro‐inflammatory cytokine production and airway concentrations of IL‐4 and IL‐13.^21^ There are suggestions that an increased count of mast cells in tissues is found in patients those patients that had low levels of vitamin D. This is explained by the vitamin D ability to increase apoptosis or inhibit the maturation of precursor cells in mice.^22^ Previous prospective studies on levels of vitamin D and asthma were analyzed in a systematic review, discovering that measured vitamin D levels in the umbilical cord at birth or through pregnancy had no associations with asthma in children later in life. Regardless, higher levels of vitamin D were linked with a lower risk of asthma progression.^23^ Reviews of observational studies which involved 23 studies enrolling 13,160 participants concluded that children who had asthma had a lower vitamin D level than healthy children.^24^ There is evidence that vitamin D levels might correlate with lung function in patients with asthma, possibly due to a lower level of IL‐13 in the airway.^21^, ^25^

Vitamin D levels in serum are influenced by various environmental aspects like diet, usage of supplements, climate, therefore its use as a single biomarker for different populations is questionable. To evaluate different populations Mongolian and Taiwanese cohorts were chosen due to latitudinal and ethnic differences. The study found that lower serum vitamin D levels (<40 ng/ml) and FokI (rs2228570) GG genotype, were associated with a higher risk of bronchial asthma in the Taiwanese. Vitamin D levels were determined to have a stronger effect on bronchial asthma than *VDR* polymorphism. In the Mongolian population, vitamin D levels were discovered to be lower (<20 ng/ml), however, no associations between VDR alterations and asthma were found.^26^ A different study of children in the Southeast region of Brazil concluded that the FokI genotype AA was a factor for the cause of asthma development, while GG genotype acted as a protecting factor. There was no insufficiency for vitamin D, therefore, no associations between vitamin D levels and VDR variants were revealed.^27^ Also, the study in the Kurdistan region determined that lower levels of vitamin D were linked with asthmatic patients. The FokI CC genotype was abundant in asthmatic patients compared to healthy individuals and associated with asthma development.^28^

A paper of the Chinese Han cohort revealed that people carrying SNPs in the VDR gene (FokI, genotype TT) had lower vitamin D levels than people with TC and CC genotypes.^29^ On top of that, the study in the Turkish Cypriot population found that individuals carrying the FokI polymorphism C>T had significantly lower levels of vitamin D when compared with others.^30^ In addition, the study in Egypt observed differences in FokI variations between the patient and control group. The TT genotype was found more frequently in the patient cohort whereas CC genotype was found more frequently in the healthy cohort. Furthermore, vitamin D levels were lower for asthmatic patients and were associated with FokI genotypes.^31^

Another paper of the Turkish children described a significant link between TaqI (CC), ApaI (CA) polymorphisms, and asthma risk. However, there were no associations between VDR polymorphisms and vitamin D levels.^32^ A different report in the Turkish Cypriot adolescent population showed that TaqI homozygous minor genotype of the VDR gene was linked with asthma. Vitamin D levels were observed to be lower in asthmatics than in healthy individuals. There were no associations found between VDR genetic variants and vitamin D levels, however, it was noticed that the TT genotype of TaqI polymorphism was more common for asthma patients with normal range vitamin D levels when comparing to the controls.^33^ The study in the Chinese population found that the frequency of ApaI (rs7975232) CC genotype was lower in the observation group, while the ApaI AC and AA genotypes were more abundant compared to healthy controls. The correlation analysis of the VDR SNVs displayed that the ApaI was negatively associated with lung function in children with bronchial asthma. Also, serum vitamin D level was lower than in the control cohort.^34^

The BsmI (rs1544410), TaqI (rs731236), and ApaI were investigated in the study of the Turkish Cypriot population where no association was found between VDR polymorphisms SNVs and serum vitamin D levels.^30^ Moreover, the study of the Turkish Cypriot adolescent population also found no significant associations between BsmI and ApaI genotypes or allele frequencies in patients and healthy groups.^33^ Furthermore, the study in the Chinese population found no statistically meaningful difference between the two groups in comparison with the base frequency of BsmI. No correlation was observed for BsmI with bronchial asthma patients' lung function levels.^34^

Another study on vitamin D levels, VDR polymorphisms, and asthma was performed in Salvador, Northeast Brazil children. VDR gene rs9729 C allele was found to be negatively correlated with asthma and vitamin D shortage. Other VDR variants, such as rs2189480 (A allele) or rs4328262 (G allele), were associated with severe forms of asthma, serving as a protector and risk factor respectively. Another VDR variant—rs10875694 (A allele) was found to be linked with atopy, despite that, no differences in vitamin D production were reported. Regarding vitamin D insufficiency, the VDR rs59128934 G allele was associated with a higher risk of insufficiency. The rs59128934 is located in an intronic region on the VDR gene, however, it is thought that this SNV might decrease vitamin D levels, which could lead to asthma. In a total six variants in the VDR gene have been shown to reduce the risk of vitamin D insufficiency: rs7967152 (A), rs9729(C), rs739837(G), rs11168287(G), rs7963776(G), and rs4237855(G). While other three VDR variants were associated with increased risk of vitamin D insufficiency: rs59128934(G), rs7965274(T), and rs2853564(C).^35^

### 2.2 VDR SNPs and vitamin D levels in allergic rhinitis patients

There are controversies in reports on allergic rhinitis (AR) and vitamin D levels, therefore Tian and Cheng^36^ in their review present the inconsistency of the results. Some studies report that higher levels of vitamin D are related to an increased risk of AR, however, other studies report the exactly opposite.^36^ The mechanisms of anti‐inflammation of vitamin D in AR resemble like previously described mechanisms in asthma.^21^, ^36^ However, it is known that vitamin D induces a switch from T helper (Th) 1 to Th2 and this leads to the development of AR.^21^, ^36^

The study from the US mother–child pre‐birth cohort reported results showing that vitamin D intake, until the second trimester, is linked with the 20% lower odds of ever developing AR. However, there were no relationship between maternal vitamin D intake or vitamin D levels at any time point with ever AR.^37^ Another study focused on determining the prevalence of vitamin D deficiency in patients diagnosed with AR. The study focused on the possible role of oral vitamin D in the efficacy of intranasal steroid spray for the treatment of AR. The researchers found that vitamin D deficiency in patients was very common (83%) and that there was a significantly better response to the treatment in the group that received vitamin D spray.^38^ In a review performed in 2016 by Kim et al.,^39^ authors investigated 19 studies and concluded that vitamin D levels were not associated with developing AR, but lower levels of vitamin D were associated with higher AR incidence in children only. A study found that the mean serum vitamin D levels in children with AR were lower than in the control cohort. However, there were no associations between vitamin D levels and the duration or severity of AR.^40^ Despite that, a study from China found that patients with constant AR exhibited a tendency of lower levels of vitamin D compared with the level in healthy individuals.^41^

To our knowledge, currently, no studies are focusing on both levels of vitamin D or vitamin D receptor SNPs in patients with AR.

### 2.3 VDR polymorphism and levels of vitamin D in atopic dermatitis patients

Most of the review articles on vitamin D and atopic dermatitis (AD) in children conclude that both clinical and epidemiological evidence indicates a positive role of vitamin D in AD. Besides, an indication was made that the AD population has lower vitamin D levels, in particular—kids diagnosed with AD.^42^, ^43^ It is thought that vitamin D can decrease pro‐inflammatory cytokines, suppress the production of IgE by B cells, and increase the level of antimicrobial peptides, for example, cathelicidin and β‐defensin and promote antimicrobial activity in AD.^44^, ^45^

The study by Su et al.^46^ found that level of vitamin D was lower in more severe AD cases compared with patients with mild ones. It suggested that lower vitamin D levels are negatively related to the severity of AD in children.^46^ Another study performed in the children cohort found that there was a link between vitamin D and AD occurrence; however, there were no associations between vitamin D and the severity of AD.^47^ Furthermore, another study in Egypt described a meaningful relationship between vitamin D deficiency and AD in children.^48^

According to data from 11 studies, vitamin D levels were lower in patients with AD.^44^ Moreover, a systematic review published in 2018 showed that lower vitamin D levels were related with the severity of AD in children.^49^

The case–control study by Kılıç et al.^50^ analyzed VDR polymorphisms such as FokI, BsmI, ApaI, and TaqI. Their findings suggested that the Bsml polymorphism is responsible for the increased probability of AD in Turkish, while FoqI, TaqI, and ApaI polymorphisms showed no correlation with AD susceptibility.^50^ There is a lack of studies focusing on VDR polymorphisms and their relationship with vitamin D levels and AD.

The summary of the association between atopic diseases and common single‐nucleotide variations of the VDR gene and vitamin D level is presented in Table 1.

**Table 1:** Atopic conditions' association with the *VDR* gene's common single‐nucleotide variations and lower vitamin D levels in patients' serum

Condition	*VDR* gene SNP^a^ association with the condition	Lower vitamin D level association with the condition	References
Asthma	Yes	Yes	Munkhbayarlakh et al.^26^, Nasiri‐Kalmarzi et al.^28^, Mohamed and Abdel‐Rehim^31^, Galvão et al.^35^
	Yes	No^b^	Rodrigues Simões et al.^27^, Kilic et al.^32^
	No	Yes	Jia et al.^29^, Tuncel et al.^30^
Allergic rhinitis	Yes	Yes	–
	Yes	No^b^	–
	No	Yes	Velankar et al.^38^, Kim et al.^39^, Dogru and Suleyman.^40^, Wu et al.^41^
Atopic dermatitis	Yes	Yes	–
	Yes	No^b^	Kılıç et al.^50^
	No	Yes	Hattangdi‐Haridas et al.^43^, Su et al.^46^, D'Auria et al.^47^, Mohamed et al.^48^

Abbreviations: SNPs, single‐nucleotide polymorphisms; VDR, vitamin D receptor.

^a^ The most commonly studied VDR gene SNPs such as FokI, TaqI, or ApaI. For more details please see the manuscript.

^b^ No data reported or no association with vitamin D levels were determined.

## 3 CONCLUSIONS

Here, we show that there is controversial data about the level of vitamin D and its role in atopy; however, more evidence shows a positive impact on the risk and outcomes of allergic diseases, especially AD and asthma. There is a lack of studies analyzing VDR gene polymorphisms and their link with vitamin D level and atopic diseases.

## CONFLICT OF INTERESTS

The authors declare that there are no conflict of interests.

## AUTHOR CONTRIBUTIONS

Lawrence Shih‐Hsin Wu, Lawrence Shih‐Jiu‐Yao Wang, Nikolajs Sjakste, and Brigita Sitkauskiene generated ideas. Brigita Sitkauskiene and Rasa Ugenskiene corrected the manuscript to its final version. Laura Tamasauskiene, Ieva Golubickaite, and Natalia Paramonova searched for articles. Laura Tamasauskiene and Ieva Golubickaite wrote the manuscript.
